# D-dimer testing, with gender-specific cutoff levels, is of value to assess the individual risk of venous thromboembolic recurrence in non-elderly patients of both genders: a post hoc analysis of the DULCIS study

**DOI:** 10.1007/s11739-019-02216-y

**Published:** 2019-11-05

**Authors:** Gualtiero Palareti, Cristina Legnani, Emilia Antonucci, Benilde Cosmi, Daniela Poli, Sophie Testa, Alberto Tosetto, Walter Ageno, Anna Falanga, Piera Maria Ferrini, Vittorio Pengo, Paolo Prandoni, for the DULCIS (D-dimer and ULtrasonography in Combination Italian Study) Investigators, Paolo Prandoni, Gualtiero Palareti, Benilde Cosmi, Cristina Legnani, Daniela Poli, Domenico Prisco, Emilia Antonucci, Angelo Ghirarduzzi, Maria Rosaria Veropalumbo, Maria Chiara Ugolotti, Nicoletta Erba, Valeria De Micheli, Sophie Testa, Oriana Paoletti, Steidl Luigi, Marco Donadini, Elena Rancan, Alberto Tosetto, Anna Falanga, Roberto Quintavalla, Piera Maria Ferrini, Rita C. Santoro, Francesco Orlandini, Raffaella Benedetti, Marco Cattaneo, Federico Lussana, Elena Bertinato, Roberto Cappelli, Attilia Maria Pizzini, Armando D’Angelo, Luciano Crippa, Lucia Angeloni, Roberta Bortolotti, Maria Rita Vandelli, Vittorio Pengo, Paolo Prandoni, Walter Ageno, Sophie Testa, Armando Tripodi, Davide Imberti, Marco Moia, Raffaele Pesavento, Nicola Magrini, Francesco Marongiu, Pietro Zonzin, Noemi Piaggesi, Mauro Silingardi

**Affiliations:** 1 Fondazione Arianna Anticoagulazione, Via Paolo Fabbri 1/3, 40138 Bologna, Italy; 2 Department of Angiology and Blood Coagulation, S. Orsola Malpighi University Hospital, Bologna, Italy; 3 Thrombosis Center, Dipartimento Oncologico AOU Careggi, Firenze, Italy; 4 Hemostasis and Thrombosis Center, AO Istituti Ospitalieri di Cremona, Cremona, Italy; 5 Hematology Department, San Bortolo Hospital, Vicenza, Italy; 6 Department of Medicine and Surgery, University of Insubria, Varese, Italy; 7 Thrombosis and Hemostasis Center, Department of Immunohematology and Transfusion Medicine, Bergamo, Italy; 8 Thrombosis and Hemostasis Center, Department of Internal Medicine, Parma, Italy; 9 Cardiology Clinic, Department of Cardiologic, Thoracic and Vascular Sciences, Università di Padova, Padova, Italy

**Keywords:** D-dimer, Gender, Venous thromboembolism, Vitamin K antagonist, Recurrence

## Abstract

Male patients, especially the young, are at a higher risk of recurrent venous thromboembolism (RVTE) than females. Recent scientific reports show the use of D-dimer does not help predict RVTE risk in males. In the present report, we reviewed the data obtained in the DULCIS study (main report published in *Blood* 2014), focusing on D-dimer results recorded in non-elderly patients of both genders included in the study, and their relationship with RVTE events occurring during follow-up. Using specifically designed cutoff values for positive/negative interpretation, serial D-dimer measurements (performed during warfarin treatment and up to 3 months after discontinuation of anticoagulation) in 475 patients (males 57.3%) aged ≤ 65 years were obtained. D-dimer resulted positive in 46.3% and 30.5% of males and females, respectively (*p* = 0.001). Following management procedure, anticoagulation was stopped in 53.7% of males and 69.5% of females, who had persistently negative D-dimer results. The rate of subsequent recurrent events was 1.7% (95% CI 0.5–4.5%) and 0.4% (95% CI 0–2.5%) patient-years in males and females, respectively, with upper limits of confidence intervals always below the level of risk considered acceptable by international scientific societies for stopping anticoagulation (< 5%). In conclusion, using sensitive quantitative assays with specifically designed cutoff values and serial measurements during and after discontinuation of anticoagulation, D-dimer testing is useful to predict the risk of RVTE and is of help in deciding the duration of anticoagulation in both male and female adult patients aged up to 65 years.

**Electronic supplementary material:**

The online version of this article (10.1007/s11739-019-02216-y) contains supplementary material, which is available to authorized users.

## Introduction

A first venous thromboembolic (VTE) event, presenting as deep vein thrombosis (DVT) and/or pulmonary embolism (PE), tends to recur after anticoagulation treatment is stopped. However, the risk of recurrence is not the same in all patients and varies greatly in relation to the nature of index event (unprovoked, or associated with permanent or transient risk factors), and to the characteristics of patients [1]. Males have consistently been found to be at a higher risk of recurrent VTE than females [2–7].

D-dimer testing, performed during or after cessation of anticoagulant treatment, has been proposed as a means to help assess the risk of recurrence in adult outpatients with unprovoked or minimally provoked VTE, which is higher in cases with positive D-dimer [4, 8, 9]. However, in other studies negative D-dimer results failed to reliably predict the risk of recurrence in male patients, being associated with a higher than acceptable rate of recurrence in these patients [3, 10]. In these results, D-dimer testing was excluded from a prediction model (the HERDOO2, [3]), or considered of limited value to be used in men, for making a decision on the duration of anticoagulation after a first unprovoked VTE [10, 11].

In the present report we re-analyzed the data recorded in the DULCIS study [12], with a view to assessing whether or not D-dimer results can be of help to guide decisions on the duration of anticoagulation in male and female patients. The prevalence of negative/positive D-dimer results in male or female patients aged 65 years or less included in the DULCIS was calculated, as well as the predictive value of D-dimer results for recurrent events in patients who did not resume anticoagulation because of persistently negative D-dimer results or because they refused to do so notwithstanding the occurrence of positive D-dimer results. For several reasons, we decided to limit our analysis to patients aged up to 65 years. It is well known, in fact, that D-dimer levels increase physiologically in elderly patients [13] which is why reliable cut-off values to be used for prediction of the risk of recurrent VTE events are currently not available. Furthermore, it has been shown that a prediction model that included D-dimer levels had a low discriminative ability in patients aged > 65 years whose risk of recurrence was reported to be the same, regardless of D-dimer levels [14]. We believe that the decision on prolonged anticoagulant treatment in the elderly should be driven by the individual perceived risk/benefit ratio, paying more attention to the risk of bleeding than to the estimated risk of VTE recurrence.

## Materials and methods

### The DULCIS study

As detailed elsewhere [12], DULCIS was a multicenter prospective study involving a cohort of 1010 patient of both sexes, aged ≥ 18 years, with a median age (IQR) of 67 (51–77) years, who had suffered a first VTE (proximal DVT of the legs, PE, or both) objectively diagnosed by compression ultrasonography of deep leg veins, computed tomographic pulmonary angiography or ventilation/perfusion lung scan, as appropriate. This trial was registered at http://www.clinicaltrials.gov as NCT00954395. Patients were eligible if the index event was either idiopathic or associated with weak risk factors (the list of weak or strong risk factors is reported in Table 1 Supplementary), and if they had completed at least 3 months of therapy with a vitamin K antagonist (VKA)—the only oral anticoagulant drug available at the time for long-term VTE treatment—with a target International Normalized Ratio (INR) of 2.5 (range 2.0–3.0 INR). The screened patients were examined to assess presence of inclusion/exclusion criteria (listed in Table 2S), with a view to avoiding inclusion of patients who were true candidates for short or extended anticoagulation, according to the available international guidelines [15].

Before inclusion, and after at least 3 months of anticoagulation, all patients received a bilateral compression ultrasonographic (CUS) examination of the proximal deep veins. Patients with detected residual vein thrombosis (RVT; > 4-mm vein diameter at probe compression in the transverse section) were invited to complete a total of 12 months VKA therapy before inclusion in the study.

### D-dimer assessment

D-dimer, the final degradation product when plasmin degrades the crosslinked fibrin, is interpreted as a marker of coagulation activation. The measurement of D-dimer plasma levels has been validated for the exclusion of VTE in symptomatic patient populations and in the diagnosis and monitoring of coagulation activation in disseminated intravascular coagulation. More recently, D-dimer assays proved clinically useful in the prediction of recurrent VTE and risk stratification of patients for VTE recurrence [16]. To this end, D-dimer plasma levels have been measured either during anticoagulant treatment [3] and/or after its discontinuation [4, 8, 9, 12]. D-dimer results have also been included in risk assessment scores proposed to predict the individual risk of recurrence after unprovoked VTE [5, 6].

For the present report, D-dimer levels were assessed using the quantitative assay routinely used in each participating center, provided it was one of those listed in Table 1; the table also shows corresponding cutoff values used to define positive or negative results in males or females aged 70 years or less (the patients involved in the present analysis were aged up to and including 65 years). Age- and sex-specific cutoff values were defined on the basis of a previous study [17], often differing from those recommended by the manufacturers for VTE exclusion and generally being lower in males than in females. Recurrent VTE and death caused by VTE were the main study outcomes, after evaluation by a central adjudication committee whose members were unaware of patient name, D-dimer testing results at inclusion, management, or enrolling center.

**Table 1:** Sex-specific cutoff levels for different D-dimer assays adopted in the DULCIS study for patients aged 70 years or less

Commercial D-dimer assay (manufacturer)	Males	Females	Cutoff values currently recommended by manufactures for VTE exclusion
VIDAS D-dimer Exclusion (bioMerieux), ng/mL	490	600	500
Innovance D-dimer (Siemens), mg/L	0.500	0.550	0.500
HemosIL D-dimer (Werfen), ng/mL	205	225	230
HemosIL D-dimer HS (Werfen), ng/mL	170	215	230
STA Liatest D-dimer (Diagnostica Stago), μg/mL	0.340	0.450	0.500

The included patients underwent serial D-dimer assessment starting at baseline during anticoagulation (T0). Patients with positive baseline D-dimer (the criteria are indicated below) were recommended to continue anticoagulation, whereas those with negative D-dimer were asked to stop VKA and repeat D-dimer testing after 15, 30, 60 and 90 days after baseline; patients were also told that at first positive D-dimer result, oral anticoagulation resumption would be recommended. The study protocol was approved by the local ethics committees and was conducted in accordance with the Declaration of Helsinki. Written informed consent was collected according to local practice.

### Statistical analysis

Descriptive analysis, expressed as median and interquartile range, was used. Differences between groups were assessed using the *X*^2^ test with Yates’ correction for categorical variables and the Mann–Whitney *U* test for continuous variables. Incidence rates of adverse events were calculated both as the number of events per 100 patients examined and the number of events per 100 patient-years (% patient-year) of observation. Kaplan–Meier survival curves were plotted to estimate the cumulative incidence of symptomatic recurrent VTE. A univariate Cox regression analysis was conducted to evaluate the relationship between the recurrent events in males and females and positive or negative D-dimer results; hazard ratios (HR) and their 95% CIs were calculated. The data were analyzed with the use of Prism software (Version 3.0, GraphPad Software Incorporated, San Diego, CA) and SPSS software (version 11.0 SPSS Inc., IBM, Armonk, NY).

## Results

The present analysis re-examined only the data of non-elderly patients [aged up to 65 years, median age 44 years (IQR 36–51)—totaling 475 with 272 males (57.3%)]—included in the DULCIS study [12] after a first symptomatic VTE episode. Table 2 shows patient profiles. Women were significantly younger than males (*p* < 0.0001), with an index event that was more often an isolated PE (*p* = 0.001). The large majority of males (88.2%) had an event that was idiopathic, whereas most females (69.0%, *p* < 0.0001) presented events that were associated to a weak risk factor, mainly hormonal contraceptive therapy (HCT, 59.1%). Males frequently had greater persistent residual vein thrombosis than females (RVT, 13.6% vs 3.0%, *p* = 0.0001), and more often received anti-platelet treatment (5.9% vs 1.0%, *p* = 0.006).

**Table 2:** Baseline characteristics of the 475 study patients

	Male	Female	*p* value
*N* (%)	272 (57.3)	203 (42.7)	
Age (years), median (IQ)	54 (45–61)	43 (34–51)	< 0.0001
Type of VTE, *n* (%)			
DVT	147 (54.0)	95 (46.8)	0.121
DVT + PE	69 (25.4)	38 (18.7)	0.084
Isolated PE	56 (20.6)	70 (34.5)	0.001
Type of risk factors, *n* (%)			
Idiopathic	240 (88.2)	63 (31.0)	< 0.0001
Weak risk factors	32 (11.8)	140 (69.0)	< 0.0001
Minor general, laparoscopic, or arthroscopic surgery	1	3	
Pregnancy or puerperium	0	4	
Hormonal contraceptive/replacement therapy	0	120/3	
Long travel	5	6	
Minor trauma, leg injury, reduced mobility	19	13	
Hospitalization in a medical ward	8	7	
Duration of previous anticoagulation, *n* (%)			
≤ 6 months	89 (32.8)	69 (34.0)	0.784
7–12 months	147 (54.0)	112 (55.2)	0.795
> 12 months	36 (13.2)	22 (10.8)	0.429
Total duration of follow-up for all patients (years)	435	348	
Follow-up (years), median (IQ)	1.95 (1.30–2.00)	2.00 (1.51–2.00)	0.048
Patients censored during follow-up, *n* (%)	23 (8.5)	7 (3.4)	0.024
Lost to follow-up, *n* (%)	1 (0.4)	1 (0.5)	0.871
Presence of RVT (> 4 mm), *n* (%)	37 (13.6)	6 (3.0)	0.0001
Associated antiplatelet treatment, *n* (%)	16 (5.9)	2 (1.0)	0.006

*DVT* proximal vein thrombosis, *IQR* interquartile range, *PE* pulmonary embolism, *RVT* residual vein thrombosis, *VTE* venous thromboembolism

**Fig. 1:**
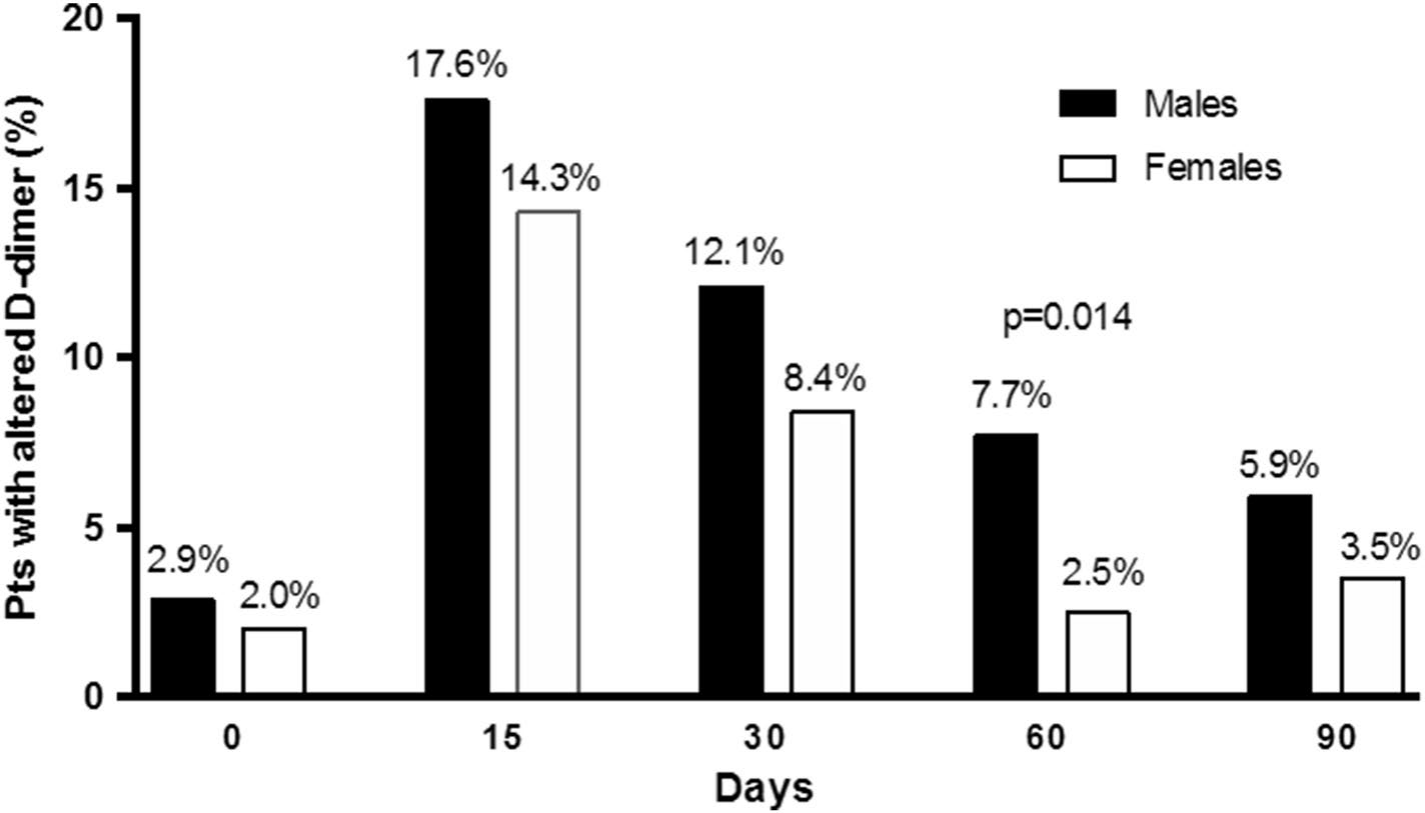
Prevalence of first-time-ever positive D-dimer results in males/females at the serial measurement days after anticoagulation withdrawal (the percentages are calculated vs the total number of patients included)

​ After serial assessment, D-dimer testing resulted positive in 188 patients (39.6%) and negative in the remainder. Altogether, D-dimer assay was positive in 126 (46.3%) males and in 62 (30.5%) females (*p* = 0.001) (Table 3). The prevalence of first-time-ever positive D-dimer results in males and females at serial measurement during and after anticoagulation withdrawal is shown in Fig. 1. The peak of the first positive result occurred at day 15 test. Positive results were more frequent in men than in women at all time-points, though the difference reached statistical significance only at 60-day control (*p* = 0.014). A late first positive D-dimer result was recorded (at T60 or T90 serial assessment) in 37 males and 12 females (29.4% and 19.3% of all patients with positive results, respectively). In women, but not in men, age was significantly higher in subjects with positive D-dimer results (*p* = 0.001). Both in men and women, the rates of positive D-dimer were not statistically different whether the index event was idiopathic or associated with WRF (males: idiopathic = 46.6%, WRF = 43.7%; females: idiopathic = 36.5%, WRF = 27.8%; differences not statistically significant).

**Table 3:** Baseline characteristics of the patients with negative/positive D-dimer results

	Males (*n* = 272)		*p* value	Females (*n* = 203)		*p* value	*p* value for positive D-dimer in males vs females
	Negative D-dimer	Positive D-dimer		Negative D-dimer	Positive D-dimer		
*n* (%)	146 (53.7)	126 (46.3)		141 (69.5)	62 (30.5)		0.001
Age (years), median (IQR)	53 (45–61)	55 (48–62)	0.120	40 (32–48)	48 (39–55)	0.001	< 0.0001
Type of VTE, *n* (%)							
DVT	82 (56.2)	65 (51.6)	0.449	70 (49.6)	25 (40.3)	0.222	0.146
DVT + PE	35 (24.0)	34 (27.0)	0.571	24 (17.1)	14 (22.6)	0.357	0.517
Isolated PE	29 (19.8)	27 (21.4)	0.745	47 (33.3)	23 (37.1)	0.601	0.022
Type of risk factors, *n* (%)							
Idiopathic	128 (87.7)	112 (88.9)	0.760	40 (28.2)	23 (37.1)	0.218	< 0.0001
WRFs	18 (12.3)	14 (11.1)	0.760	101 (71.6)	39 (62.9)	0.218	< 0.0001
(Total HCT *n* = 120; *n*; %)				(89; 74.2^a^)	(31; 25.8^a^)	0.081	
Total duration of follow-up for all patients (years)	234	201		241	107		
Patients censored during follow-up, *n* (%)	11 (7.5)	12 (9.5)	0.554	3 (2.1)	4 (6.4)	0.121	0.474
Lost to follow-up, *n* (%)	1 (0.7)	0 (0)	0.348	0 (0)	1 (1.6)	0.133	0.156
Presence of RVT (> 4 mm), *n* (%)	18 (12.3)	19 (15.1)	0.502	3 (2.1)	3 (4.8)	0.294	0.039
Associated antiplatelet treatment, *n* (%)	10 (6.8)	6 (4.8)	0.485	2 (1.4)	0 (0)	0.350	0.080

^a^These percentages are calculated versus the 120 women whose VTE event occurred during HCT

*DVT* proximal vein thrombosis, *HCT* hormonal contraceptive therapy, *IQR* interquartile range, *PE* pulmonary embolism, *RVT* esidual vein thrombosis, *VTE* venous thromboembolism, *WRFs* weak risk factors

All patients with negative D-dimer results (n. 287) were advised to stop anticoagulant treatment, whereas those with positive results (n. 188) were recommended to continue or resume anticoagulation with a VKA (warfarin in all cases), at the time the only type of oral anticoagulant drug available. Five recurrent VTE events occurred in 2-year follow-up in patients who had persistently negative D-dimer results (Table 4), with an incidence of 1.7% patient-years (95% CI 0.5–4.5) in males and 0.4% patient-years (95% CI 0–2.5) in females. In contrast, 8 recurrent events occurred among the 59 patients who refused to resume anticoagulation after positive D-dimer results, with an incidence of 7.3% patient-years (95% CI 2.8–16.5) and 11.5% patient-years (95% CI 3.2–29.8) in males and females, respectively. Of the 8 recurrent events, 4 occurred in patients whose first positive D-dimer result was obtained at T15, 3 at T30 and one event in a patient with positive D-dimer at T60.

**Table 4:** Clinical events occurred during follow-up in the investigated patients

	Negative D-dimer		Positive D-dimer, without anticoagulation		Positive D-dimer, with anticoagulation	
	Male *n* = 146	Female *n* = 141	Male *n* = 44	Female *n* = 15	Male *n* = 82	Female *n* = 47
Duration of follow-up (years)	234	241	68	26	133	81
VTE recurrences during follow-up, n (%; 95% CI) (in women with HCT-associated index event)	4 (2.7; 0.8–7.1)	1 (0.7; 0–4.3) (1/89)	5 (11.4; 4.5–24.4)	3 (20.0; 6.3–46.0) (1/11)	1 (1.2; 0–7.2)	0 (0/20)
Incidence per 100 pt-y, % (95% CI)	1.7 (0.5–4.5)	0.4 (0–2.5)	7.3 (2.8–16.5)	11.5 (3.2–29.8)		
Type of event, *n*						
DVT	3	1	5	1	0	0
PE	1	0	0	1	1	0
DVT + PE	0	0	0	1	0	0
Other outcomes, *n*						
Death^a^	0	0	0	0	0	0
Isolated distal DVT	2	1	2	0	0	0
SVT	4	1	1	0	1	0
Arterial vascular event	1	0	0	0	0	0
Major bleeding, *n* (%; (95% CI)	0	0	0	0	2 (2.4; 0.1–9.0)	1 (2.1; 0–12.1)
Incidence per 100 pt-y (95% CI)					1.5 (0–5.7)	1.2 (0–7.3)

*DVT* proximal deep vein thrombosis, *PE* pulmonary embolism, *SVT* superficial vein thrombosis

^a^No death could be attributed to thrombotic event

**Fig. 2:**
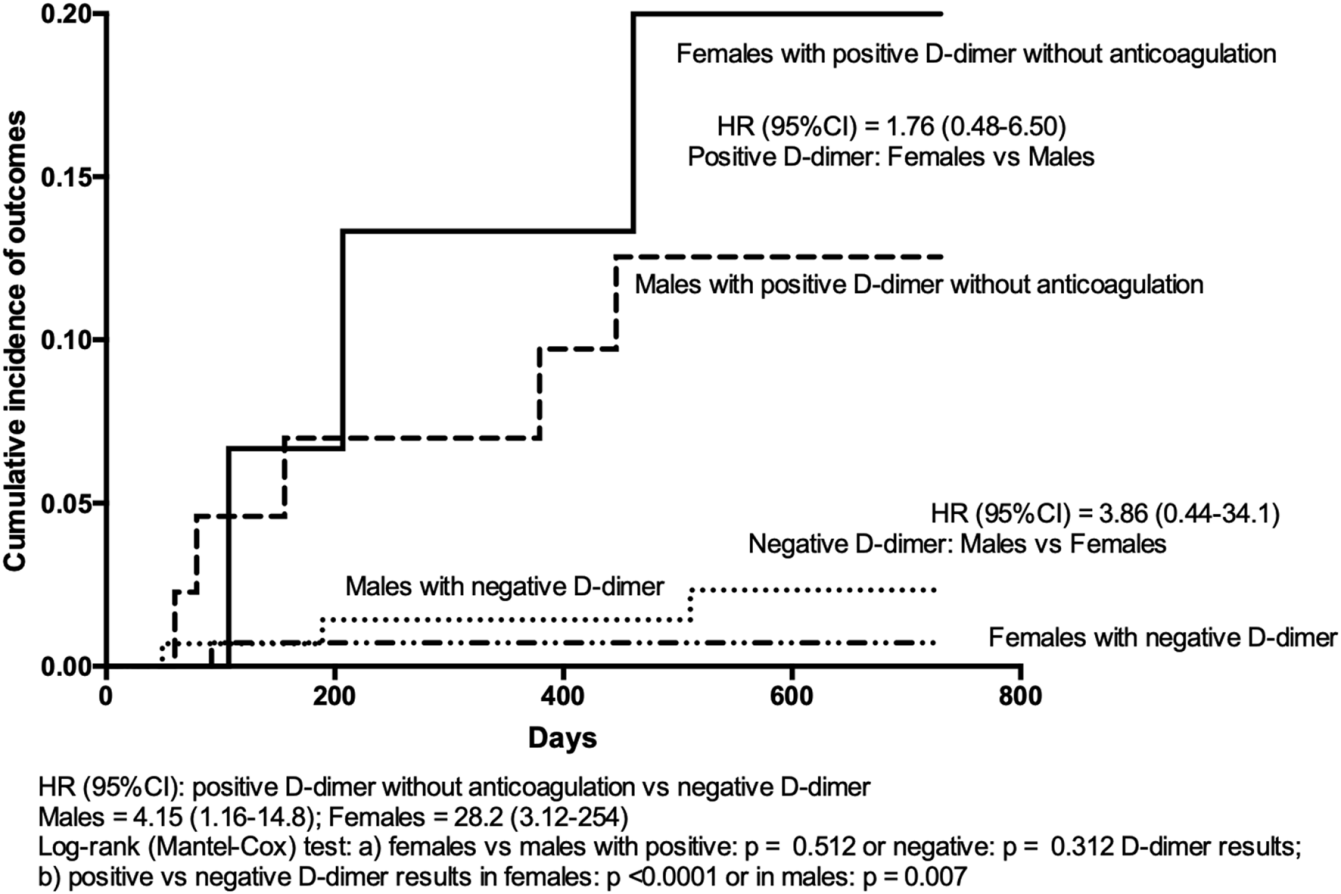
Kaplan–Meier cumulative event rates for the primary efficacy outcome in males/females with persistently negative D-dimer results in whom anticoagulation was definitively stopped and in males/females with positive D-dimer results who refused to resume anticoagulation

In women with an index event that was HCT-related, one recurrent event occurred among the 89 with negative D-dimer, and one among the 11 with positive results who stayed without anticoagulation. The Kaplan–Meier curves of cumulative primary efficacy outcomes in males/females with persistently negative D-dimer results stopping anticoagulation and positive D-dimer results refusing to resume anticoagulation are shown in Fig. 2. Hazard ratios for occurring outcomes were significantly higher, though with wide confidence intervals, in males and females with positive D-dimer results than in corresponding subjects with negative results [HR 4.15 (95% CI 1.16–14.8), and 28.2 (95% CI 3.12–254), respectively]. Vice versa, HR was not significantly different between male or female subjects with positive [1.76 (95% CI 0.48–6.50)] or negative [3.86 (95% CI 0.44–26.1)] D-dimer results (Fig. 2).

## Discussion

The present report shows that in non-elderly patients (aged up to 65 years) with VTE either unprovoked or associated with weak risk factors, serial D-dimer measurements can reliably be used to assess individual risk of recurrence and manage the duration of anticoagulation irrespective of gender. All patients with positive D-dimer results were encouraged to resume anticoagulation so as to prevent recurrent events and reduce the number of subjects with repeatedly negative D-dimer in whom the rate of recurrent VTE was found to be very low in both genders. Indeed, anticoagulation was safely stopped in 53.7% of males and 69.5% of females due to negative D-dimer results. The incidence of subsequent recurrence was very low: 1.7% and 0.4% patient-years in males and females, respectively, the upper limits of 95% confidence intervals being < 5% (a level of risk that is considered acceptable to stop anticoagulation) [18] in both genders.

Our D-dimer procedure resulted more frequently positive in men than in women. Besides the significantly older age of males, we believe this result can mainly be accounted for by the lower D-dimer cut-off levels adopted in our study in males versus females. This approach was intended to identify more males at potentially higher risk of recurrent VTE and extend anticoagulation in them. There is, in fact, general agreement that males are at a higher risk of recurrent VTE events than females [2–4, 19]. Male sex is included as a relevant risk factor in virtually all algorithms designed to identify individual risk of recurrent VTE [5, 6]. One prediction rule, incorporating D-dimer testing only during anticoagulation, concluded that the proposed rule was useless in men since all needed indefinite anticoagulation [7]. The first-time-ever positive D-dimer results were more prevalent in men than in women at all serial measurements with a difference that reached statistical significance at T60, while the rate of first positive results dropped dramatically in women (see Fig. 1). The fact that males, more than females, may present signs of hypercoagulability at a later date, a condition possibly associated with a risk of VTE recurrence, confirms the importance of a serial D-dimer determination, especially in men.

The performance of D-dimer testing in our study as predictor of risk for VTE recurrence was very good, helping to distinguish subjects at low or high risk in both women and in men. The good results obtained with our D-dimer procedure are probably due to the cutoff values we adopted, values which in males were generally lower than those recommended by manufacturers for diagnostic procedures and lower than those used for women who physiologically have higher D-dimer values than men [5, 20]. As a consequence, more male (46.3%) than female (30.5%, *p* = 0.001) patients had positive D-dimer results and resumed anticoagulation with subsequent protection against recurrence risk.

Another factor informing the good results obtained with our procedure was serial D-dimer measurement which, following a previous study of our group [21], covered the first three months after anticoagulation suspension. Repeated D-dimer measurement allowed detection of several patients developing signs of hypercoagulability very early (T15) or later than 30 days (T30, that is the generally used timing assessment [4]) after anticoagulation interruption. In fact, 29.4% of males and 19.5% of female patients had their first positive D-dimer results 60 or 90 days after anticoagulation was interrupted. Furthermore, among the patients who refused to resume anticoagulation, recurrent VTE occurred in 3 patients with a first positive D-dimer at T15 and in one with positive D-dimer at T60. These results confirm the importance of serial D-dimer assessment in helping to reduce the risk of recurrence either early after anticoagulation is stopped or in subjects who present late signs of hypercoagulability.

The index event was unprovoked in the vast majority of males (88.2%), but only in 31.0% of women (*p* < 0.0001). It seems reasonable to ascribe this difference to the effect of the large number of women (n. 120) included for HCT-related index event. The prevalence of positive D-dimer results was not statistically different in males and females whose index event was either unprovoked a or associated with weak risk factors. These results demonstrate that a condition of hypercoagulability may occur after anticoagulation is stopped in subjects of both sexes whose index event was either idiopathic or WRF-related and that D-dimer testing helps to identify these subjects. In particular, it can be noticed that among the 120 women included in the study following HCT-related VTE event, a condition generally regarded as being at low risk of recurrence [6, 22], our D-dimer procedure allowed identification of some subjects (about 25%) with positive testing in whom the risk of recurrence was non-negligible as shown by the single PE recurrence case that occurred during follow-up among the 11 with positive D-dimer who did not resume anticoagulant therapy.

In contrast with our results, other studies have not found negative D-dimer results safe enough for stopping anticoagulation, especially in men. Rodger et al. [3] assessed D-dimer levels while patients were still on warfarin, using the quantitative Vidas D-dimer reagent (bio-Mérieux) with cutoff level for positive results set at ≥ 250 ng/mL (half the value adopted for VTE exclusion with that assay). They found that positive D-dimer results were associated with higher recurrence risk in women but not in men. D-dimer assessment performed only while patients were on anticoagulants makes the results of this study hard to compare with those of all other studies on the issue. It is reasonable to surmise that a potential condition of hypercoagulability (and higher D-dimer levels) is effectively counteracted with effective anticoagulation but may come back in play when anticoagulants are stopped. This effect is clearly shown in Fig. 1 of the present report, where it can be seen that the rate of positive D-dimer is very low during anticoagulation but increases sharply at the following time-points, till 60 days and more after anticoagulation was interrupted.

More recently, Kearon et al. [10] assessed D-dimer levels twice, once during anticoagulation and the other one month after its withdrawal, in a cohort of 410 patients who had a first unprovoked VTE event. They used a qualitative assay (Clearview Simplify, by Alere), a point-of-care assay intended to give positive or negative results via calibration versus diagnostic level for VTE exclusion. Anticoagulant therapy was definitively stopped in 319 patients after 2 negative D-dimer results; their rate of recurrent VTE was 6.7% (95% CI 4.8% to 9.0%) per patient-year, higher in men (9.7% (CI 6.7–13.7%). The authors concluded that “The risk for recurrence in patients with a first unprovoked VTE who have negative D-dimer results is not low enough to justify stopping anticoagulant therapy in men…”. We believe that these disappointing results were mainly due to the extremely low rate of positive results (only 16.8% of the whole patient cohort), with a subsequent high proportion of patients not resuming anticoagulation and a high rate of VTE recurrence in these patients. In contrast, most studies on the issue have pointed to a much higher rate of patients with positive D-dimer results. Post-anticoagulation D-dimer results were positive in 45.4% of patients in a patient-level meta-analysis [4], and in 36.5% of patients in the present analysis involving patients aged up to 65 years. The qualitative D-dimer assay used in Kearon’s study (Clearview Simplify) was the same as the one we used in the PROLONG study published in 2006, in which the rate of positive results was more than twice (36.7%) that recorded in Kearon’s study [8]. However, the manufacturers of D-dimer assays used in the studies were different: Alere in the Kearon’s study and Inverness Medical Professional Diagnostics in the PROLONG study. A different sensitivity of the two assays cannot be excluded, and was probably lower in the case of Alere assay, thus explaining the particularly high number of patients with negative D-dimer results in the Kearon study and the subsequent relatively high incidence of recurrent events in the patients who did not resume anticoagulant treatment.

Our study has several limitations. Firstly, our conclusions can only be applicable in individuals aged 65 years or less. Since D-dimer levels increase sharply with age [13], it is very difficult to define age-appropriate cutoff values and to identify possible different risks of recurrence among older individuals, as confirmed in the recent validation study of the DASH score which failed to be risk-predictive in elderly patients [14]. Furthermore, it should not be forgotten that some elderly VTE patients may be at a higher risk of bleeding while on anticoagulant therapy and so may benefit from discontinuing anticoagulation after the first months irrespective of the nature of their VTE [15]. A population-based observational study [23] recorded incidence rates of major bleeding episodes of 13.2% in subjects aged ≥ 65 years versus 6.6% in those aged < 65 years, after 1 year of follow-up (*p* < 0.001), with 77% of bleeding events occurring during anticoagulation [24]. This confirmed the higher risk of bleeding in VTE subjects aged 65 years or more. Secondly, the inclusion of patients with HCT-related VTE may be questioned, as the risk of recurrent events is low provided hormonal therapy is definitively discontinued [6, 25]. We considered hormonal therapy a weak risk factor for VTE and so included these patients in the study as well as those with different weak risk factors. Moreover, since negative D-dimer findings will be markedly prevalent in this population they can be seen as a useful factor for reassuring women to stop anticoagulation, while the few with positive results can be more carefully informed of their risk and so may be in a better condition to decide what to do next. Thirdly, we are aware that serial D-dimer assessments in the first 3 months after anticoagulation interruption may be inconvenient for both patients and physicians and may generate confusion in professionals, especially in case of a suspected VTE recurrence during the period. However, we are convinced that D-dimer assessment should be extended beyond 1 month after anticoagulation is interrupted to detect patients with late onset of signs of hypercoagulability who still may have a non-negligible risk of recurrence. Finally, all the mentioned studies were performed in patients who received vitamin K antagonists as anticoagulant drug. Results of a recent study suggest that D-dimer levels can change differently, either during or after anticoagulation is discontinued, when direct oral anticoagulants are used for anticoagulation treatment in VTE patients [26].

In conclusion, the present sub re-analysis of data collected in the DULCIS study [12] suggests that in both male and female patients aged up to 65 years with a first VTE event unprovoked or associated with weak risk factors, the adoption of serial D-dimer assessment (using sensitive quantitative assays, with specifically established cutoff values to indicate negative/positive results) may help attending physicians to evaluate the individual risk of recurrence and decide whether to extend or discontinue anticoagulation.

## Electronic supplementary material

Below is the link to the electronic supplementary material.

Supplementary material 1 (DOCX 16 kb)
