# Supplementary material for: D-dimer testing, with gender-specific cutoff levels, is of value to assess the individual risk of venous thromboembolic recurrence in non-elderly patients of both genders: a post hoc analysis of the DULCIS study
Source: Intern Emerg Med. 2019 Nov 5;15(3):453–62. doi: 10.1007/s11739-019-02216-y (PMC7165144; doi:10.1007/s11739-019-02216-y)
Supplement: Supplementary file 1 — Supplementary material 1 (DOCX 16 kb) [file 11739_2019_2216_MOESM1_ESM.docx]

D-DIMER TESTING, WITH GENDER-SPECIFIC CUTOFF LEVELS, IS OF VALUE TO ASSESS THE INDIVIDUAL RISK OF VENOUS THROMBOEMBOLIC RECURRENCE IN NON-ELDERLY PATIENTS OF BOTH GENDERS: A POST-HOC ANALYSIS OF THE DULCIS STUDY

**Journal: Internal and Emergency Medicine**

^1^Gualtiero Palareti, ^1^Cristina Legnani, ^1^Emilia Antonucci, ^2^Benilde Cosmi, ^3^Daniela Poli , ^4^Sophie Testa , ^5^Alberto Tosetto, ^6^Walter Ageno, ^7^Anna Falanga, ^8^Piera Maria Ferrini, ^9^Vittorio Pengo , and ^1^Paolo Prandoni for the DULCIS (D-dimer and ULtrasonography in Combination Italian Study) Investigators

^1^Fondazione Arianna Anticoagulazione, Bologna, Italy; ^2^Department of Angiology and Blood Coagulation, S. Orsola Malpighi University Hospital, Bologna, Italy; ^3^Thrombosis Center, Dipartimento Oncologico AOU Careggi, Florence, Italy; ^4^Hemostasis and Thrombosis Center, AO Istituti Ospitalieri di Cremona, Cremona, Italy;  ^5^Hematology Department, San Bortolo Hospital, Vicenza, Italy; ^6^Department of Medicine and Surgery, University of Insubria, Varese, Italy; ^7^Thrombosis and Hemostasis Center, Department of Immunohematology and Transfusion Medicine, Bergamo, Italy; ^8^Thrombosis and Hemostasis Center, Department Internal Medicine, Parma, Italy ; ^9^Cardiology Clinic, Department of Cardiologic, Thoracic and Vascular Sciences, University of Padua

Corresponding author

Gualtiero Palareti

e.mail: gualtiero.palareti@unibo.it

Table 1 Supplementary. List of weak or strong risk factors for venous thromboembolism

1. Weak risk factors

Minor surgery

Arthroscopic, or laparoscopic general surgery

Pregnancy or puerperium

Contraceptive or replacement hormonal therapy

Long trip (> 6 h)

Minor trauma (not requiring hospitalization, plaster casting, or immobilization)

Hospitalization in a medical hospital

Reduced mobility (not complete immobilization)

B) Strong risk factors

Major surgery (within 3 mo.)
Bed resting (greater or equal to 4 d, even if with bathroom privileges)
Major trauma (within 3 mo.)
Plasters or immobilization (within 3 mo.)

More documented VTE episodes (proximal DVT and/or PE)
Active cancer or hematologic disease
Antithrombin deficiency
Antiphospholipid antibody syndrome (Sydney criteria)

Table 2 Supplementary. Criteria for inclusion/exclusion in the DULCIS study

**Inclusion criteria**

- Age 18 y or greater
- First episode of proximal DVT of lower limbs and/or PE that was: unprovoked or associated with one of following weak risk factors:
  - minor, arthroscopic, or laparoscopic general surgery
  - pregnancy or puerperium
  - contraceptive or replacement hormonal therapy
  - long trip (at least 6 h)
  - minor trauma (not requiring hospitalization, plaster casting, or immobilization)
  - hospitalization in a medical hospital
  - reduced mobility (not complete immobilization)
- Anticoagulation therapy (VKA, INR 2.0-3.0) for > 3 months
- Ability to provide informed consent

**Exclusion criteria**

- Age < 18 y
- Duration of anticoagulation < 3 months
- Inability or refusal to give consent
- Limited life expectation (< 1 y)
- Persisting increased systolic pulmonary arterial pressure (values greater than or equal to 35 mm Hg [or > 40 mm Hg if BMI > 30 or age > 75 y] estimated with echocardiography)
- Geographical inaccessibility
- Venous thrombosis in different sites (upper limbs, splanchnic veins, jugular or cerebral veins)
- Pregnancy or puerperium (first 6 weeks after birth) at the time of screening examination
- Severe renal (creatinine level > 2 mg/dL [177 mmol/L]) or liver failure (e.g., acute hepatitis, chronic active hepatitis, or cirrhosis; or an alanine aminotransferase level that was 3 times the upper limit of the normal range or higher)
- Presence of one of the following criteria for short anticoagulation
  - VTE post major surgery (within 3 mo)
  - VTE post bed resting (greater or equal to 4 d)
  - VTE post major trauma (within 3 mo)
  - VTE post plasters or immobilization (within 3 mo)
  - High bleeding risk
- Presence of one of the following criteria for extended anticoagulation
  - More documented VTE episodes (proximal DVT and/or PE)
  - Active cancer or hematologic disease
  - Antithrombin deficiency
  - Antiphospholipid antibody syndrome (Sydney criteria)
  - PE with shock or life-threatening prolonged hypotension
- Different indications for anticoagulation
- Severe cardiorespiratory insufficiency (NYHA 3 or 4)
